# Antithrombotic treatment in atrial fibrillation patients needing percutaneous coronary intervention

**DOI:** 10.1007/s12471-021-01546-x

**Published:** 2021-02-10

**Authors:** M. V. Huisman

**Affiliations:** Department of Medicine—Thrombosis and Hemostasis, Leiden University Medical Center, Leiden, The Netherlands

Patients with nonvalvular atrial fibrillation (AF) need chronic with by non-vitamin K oral anticoagulants (NOACs) or vitamin K antagonists [1]. NOACs are more convenient to use and have a larger net clinical benefit than vitamin K antagonists. While this development has simplified anticoagulant management in these patients, it is different for AF patients needing coronary interventions. It is estimated that, at some time, around 20% of patients with AF require percutaneous coronary intervention (PCI) and subsequent antiplatelet therapy.

While guidelines contain class I recommendations for the default immediate periprocedural and post-PCI antithrombotic therapy, as well as postdischarge up to 12 months, there is less certainty, about the same antithrombotic therapy regimens in patients on NOACs who have a high bleeding risk, a high ischaemic risk or both (see Fig. 8 in Collet et al. [2]).

These uncertainties are displayed in the article by De Veer and colleagues in this issue of the *Netherlands Heart Journal* [3]. In an international survey among interventional cardiologists, most of whom were working in a Dutch hospital, the authors asked about the antithrombotic management of two hypothetical patients with AF: one patient using a standard dose of a NOAC who presents with an acute non-ST-elevation myocardial infarction (NSTEMI) and requires PCI with stenting and another patient using a standard dose of a NOAC who needs to undergo elective PCI.

While the authors concluded that there was heterogeneity in the management strategy among the interventional cardiologists, reflecting the guidelines, there is another way to look at their interesting data: there are areas with certainty and agreement, where this is needed, and there are areas with disagreement and heterogeneity, where this is appropriate. An example of the first, and in line with the guidelines, was that 96% all respondents would start antiplatelet therapy as soon as possible after admission of the AF patient with NSTEMI, of which 70% would start dual antiplatelet therapy (consisting of acetylsalicylic acid and clopidogrel) and 23% would initiate monotherapy (prasugrel, ticagrelor or clopidogrel). Along the same line, at discharge, 70% would start triple antithrombotic therapy (oral anticoagulant plus two antiplatelet agents), whereas only 9% would start this treatment in patients with a high bleeding risk.

Uncertainty arose in the following circumstances: at admission, more than half (53%) would stop the NOAC; during PCI, 34% would administer a reduced dose of heparin; and at discharge, 43% would start triple antithrombotic therapy in patients with a combined high ischaemic and high bleeding risk. One of the difficulties with the latter strategy is the lack of a validated bleeding score. The PRECISE-DAPT and ARC-HBR scores [4, 5] may be difficult to apply in routine clinical practice, as several of their criteria are quite detailed and these scores have not been validated in rigorous randomised trials.

The tailoring of antithrombotic treatment based on bleeding risk deserves further comment. Several recent trials (AUGUSTUS, RE-DUAL PCI, ENTRUST-AF PCI, PIONEER-PCI) [6–9], which followed the same philosophy as the pivotal WOEST study [10], have focused on reducing bleeding. This makes sense, given that major bleeding may lead to stopping anticoagulant treatment with a subsequent increased risk of ischaemic and thrombotic complications, or even mortality [11, 12]. An important caveat, however, is that while the individual trials were powered to address the safety of the tested strategy, they were too small to reliably assess differences in ischaemic complications.

An important signal was given by a recent meta-analysis of four trials with more than 10,000 patients, in which dual antithrombotic treatment was compared with triple antithrombotic treatment in AF patients undergoing PCI [13]. The primary safety endpoint (combination of major or clinically relevant nonmajor bleeding) was lower with the dual antithrombotic than with the triple antithrombotic treatment, with a risk ratio (RR) reduction of 0.66 (95% confidence interval (CI) 0.56–0.78). This benefit was counterbalanced by a significant increase in stent thrombosis (RR 1.59, 95% CI 1.01–2.50). This translates into an absolute reduction in major bleeding of 2% and an absolute increase in stent thromboses of 0.4%, without an effect on overall major adverse cardiovascular events. Of note, a subanalysis of one of the trials (AUGUSTUS) showed that the stent thrombosis rate was highest within the first 30 days, with a similar timing for bleeding events [14].

A second consideration is choosing the proper dose of NOACs in AF patients who also receive antiplatelet therapy. Although guidelines advise to administer the lowest possible dose [1, 2], only dabigatran in a reduced dose of 110 mg twice daily was evaluated in parallel to dabigatran 150 mg twice daily [15], whereas the full dose of the other NOACs has been tested.

From this timely survey, it is clear that new and unresolved territories can and should be explored, ideally in a randomised fashion. Among these is the value of different decision rules for ischaemia and bleeding and the proper heparin dose during PCI for patients taking NOACs. Finally, even in this evidence-based era—with its multiple guidelines, many of which are already outdated the moment they appear because of new studies—there is still room for patient-tailored, balanced decision-making. In that case, the adage ‘In dubio abstine’ is valid as an aid in reducing bleeding, although clinicians should be aware that ‘too little’ antithrombotic treatment may lead to ‘too much’ ischaemia and thrombosis.
